# Supplementary material for: Analysis of Conductance Probes for Two-Phase Flow and Holdup Applications
Source: Sensors (Basel). 2020 Dec 9;20(24):7042. doi: 10.3390/s20247042 (PMC7763996; doi:10.3390/s20247042)
Supplement: Supplementary file 1 [file sensors-20-07042-s001.zip › sensors-983932-supplementary/Supplementary 1-programMATLAB_ Bloc de notas.pdf]

```
% programm conductance sensors case of Coney
% two flush mounter sensors
% New version with new recurrence relations to compute derivatives of
% Modified Bessel Functions
close all
clc
clear

% Case 3D with the sensor at z=0
% sensors located at angles (thet1+thet2)/2 and -(thet1+thet2)2
sz=0.288  % sensor heigh sz=0.2 cm (cm)
sw =0.215 % sensor wide
D=5.08  % pipe diameter D (cm)
R=D/2 % internal radius of the pipe R
a=0.1
theta1=a/R    % half distance between sensors
theta2=(a+sw)/R
NR=14
De=0.415  % distance between sensor centers De
for i=1:NR
    Rin(i)=2.08+0.03*i  % Radius of the calibration piece
    Din(i)=Rin(i)*2 % diameter of the calibration piece
    delta(i)=R-Rin(i)
end
dnor=(De-sw)/2  % normalization distance
Htop=10  % distance from z=0 (center) to top
Hbot=10 % distance from z=0 to bottom
nmaxm=100 % number of azimuthal modes
nmaxn=100 % number of radial modes
H=Htop+Hbot
ctop=Htop/dnor;
cbot=Hbot/dnor;
swnor=sw/dnor ; % snor=lambda-1, normalized wide of the sensor
lambda=1+swnor
% non symmetric case
% calculation of sum( (a(n)/n^3)fn(Rin,R))
pi=3.141592;
 for j=1:NR
    SumG(j)=0
    SumG0mmax(j)=0
    sumG0m(j)=0     % addition of contributions of terms 0m
    SumGnmmax(j)=0  % addition of contributions of terms nm  max
    SumGnm(j)=0.
 end
% computes the contribution of G0m
    C1=2*sz*sz*R*pi^3/H^3
for j=1:NR
    RatR=Rin(j)/R;
for i=1:nmaxm
    coefRat=(1+RatR^(2*i))/(1-RatR^(2*i));
    am2dm3(i)=(cos(i*theta2)-cos(i*theta1))^2/i^3;
    am2dm3rin(i)=coefRat*(cos(i*theta2)-cos(i*theta1))^2/i^3;
end
    SumG0mmax(j)=C1*sum(am2dm3);
```

```
    SumG0m(j)=C1*sum(am2dm3rin);
end
% computes the contributions of Gnmmax
for j=1:NR         % different inner radius
for i=1:nmaxm      % i=m azim modes
    am2dm2(i)=(cos(i*theta2)-cos(i*theta1))^2/i^2;
    sumanGmax(i)=0;
    sumanGmax1=0;
for k=1:nmaxn      % k=n radial modes
    gam(k)=2*k*pi/(Htop+Hbot);
    senogn23(k)=(sin(gam(k)*sz/2))^2/k^3;
    IBR(i,k)=besseli(i,gam(k)*R);
    if(i==1)
         I0R(k)=besseli(0,gam(k)*R);
         DIBR(i,k)=(I0R(k)-i*IBR(i,k)/(gam(k)*R));
    else
         DIBR(i,k)=(IBR(i-1,k)-i*IBR(i,k)/(gam(k)*R));
    end
    ratio(i,k)=IBR(i,k)/DIBR(i,k);
    Gnmmax(i,k)=am2dm2(i)*senogn23(k)*ratio(i,k);
    sumanGmax1=sumanGmax1+Gnmmax(i,k);
end
    sumanGmax(i)=sumanGmax1;
end
    SumGnmmax(j)=sum(sumanGmax);
end
% Contribution of terms Gnm
for j=1:NR
    Rin1=Rin(j)
for i=1:nmaxm      % i=m azim modes
    am2dm2(i)=(cos(i*theta2)-cos(i*theta1))^2/i^2;
    SumaGnm1=0
for k=1:nmaxn      % radial modes
    gam(k)=2*k*pi/(Htop+Hbot);
    senogn23(k)=(sin(gam(k)*sz/2))^2/k^3;
% computation of the modified bessel functions
% calculation of
fnm(gamn*R)=(Im(gamn*R)-anm*Km(gamn*R))/(I'm(gamn*R)-anm*K'm(gamn*R))
%    amn=I'm(gamn*R)/K'm(gamn*R)
%
    IBR(i,k)=besseli(i,gam(k)*R);
    KBR(i,k)=besselk(i,gam(k)*R);
    IBRin(i,k)=besseli(i,gam(k)*Rin1);
    KBRin(i,k)=besselk(i,gam(k)*Rin1);
    IBR(i+1,k)=besseli(i+1,gam(k)*R);
    KBR(i+1,k)=besselk(i+1,gam(k)*R);
    IBRin(i+1,k)=besseli(i+1,gam(k)*Rin1);
    KBRin(i+1,k)=besselk(i+1,gam(k)*Rin1);
%
    if(i==1)
         I0R(k)=besseli(0,gam(k)*R);
         K0R(k)=besselk(0,gam(k)*R);
         I0Rin(k)=besseli(0,gam(k)*Rin1);
         K0Rin(k)=besselk(0,gam(k)*Rin1);
```

```
%          DIBRin(i,k)=(I0Rin(k)-i*IBRin(i,k)/(gam(k)*Rin1));
%          DKBRin(i,k)=-(K0Rin(k)+i*KBRin(i,k)/(gam(k)*Rin1));
%          DIBR(i,k)=(I0R(k)-i*IBR(i,k)/(gam(k)*R));
%          DKBR(i,k)=-(K0R(k)+i*KBR(i,k)/(gam(k)*R));
% new1
        DIBRin(i,k)=0.5*(I0Rin(k)+IBRin(i+1,k));
        DKBRin(i,k)=-0.5*(K0Rin(k)+KBRin(i+1,k));
        DIBR(i,k)=0.5*(I0R(k)+IBR(i+1,k));
        DKBR(i,k)=-0.5*(K0R(k)+KBR(i+1,k));
% end new 1
         amn(i,k)=DIBRin(i,k)/DKBRin(i,k);
         fmn(i,k)=(IBR(i,k)-amn(i,k)*KBR(i,k))/(DIBR(i,k)-amn(i,k)*DKBR(i,k));
    else
%          DIBRin(i,k)=(IBRin(i-1,k)-i*IBRin(i,k)/(gam(k)*Rin1));
%          DKBRin(i,k)=-(KBRin(i-1,k)+i*KBRin(i,k)/(gam(k)*Rin1));
%          DIBR(i,k)=(IBR(i-1,k)-i*IBR(i,k)/(gam(k)*R));
%          DKBR(i,k)=-(KBR(i-1,k)+i*KBR(i,k)/(gam(k)*R));
          DIBRin(i,k)=0.5*(IBRin(i-1,k)+IBRin(i+1,k));
          DKBRin(i,k)=-0.5*(KBRin(i-1,k)+KBRin(i+1,k));
          DIBR(i,k)=0.5*(IBR(i-1,k)+IBR(i+1,k));
          DKBR(i,k)=-0.5*(KBR(i-1,k)+KBR(i+1,k));
         amn(i,k)=DIBRin(i,k)/DKBRin(i,k);
         fmn(i,k)=(IBR(i,k)-amn(i,k)*KBR(i,k))/(DIBR(i,k)-amn(i,k)*DKBR(i,k));
    end
         G0nm(i,k)=am2dm2(i)*senogn23(k)*fmn(i,k);
         SumaGnm1=SumaGnm1+G0nm(i,k);
end
    SumaGnm(i)=SumaGnm1;
end
    SumGnm(j)=sum(SumaGnm);
    fracliquid(j)=(R^2-Rin(j)^2)/R^2;
    voidfrac(j)=Rin(j)^2/R^2
    RatioG(j)=(SumG0mmax(j)+SumGnmmax(j))/(SumG0m(j)+SumGnm(j));
end
    zDH=Htop/H
    DeD=De/D
    SDe=sw/De
    figure (1)
    plot(fracliquid, RatioG,'x')
    title(DeD)
    xlabel('liquid fraction')
    ylabel('Gapp/Gmax')
    figure (2)
    plot(Rin,RatioG,'*')
    title(zDH)
    xlabel('Rin')
    ylabel('Gapp/Gmax')
    figure (3)
    plot(voidfrac, RatioG,'x')
    title('horizontal sensors De=4mm, sz=sw=2mm H=20 cm')
    xlabel('Voidfrac')
    ylabel('Gapp/Gmax')
    figure (4)
    plot(10*delta, RatioG,'+')
```

```
xlabel('delta mm')
ylabel('Gapp/Gmax')
greldelta=fopen('grelvsdelp6.txt','w')
Greldelta1=[10*delta;RatioG]
fprintf(greldelta,'%6.4f %6.4f\r\n',Greldelta1)
status=fclose(greldelta)
```
