# Supplementary material for: Analysis of Conductance Probes for Two-Phase Flow and Holdup Applications
Source: Sensors (Basel). 2020 Dec 9;20(24):7042. doi: 10.3390/s20247042 (PMC7763996; doi:10.3390/s20247042)
Supplement: Supplementary file 1 [file sensors-20-07042-s001.zip › sensors-983932-supplementary/Supplementary 2-Nomenclature.docx]

**Nomenclature:** **Latin letters**

| **Symbol** | **-Definition-** | **Symbol** | **-Definition-** | **Symbol** | **-Definition-** |
| --- | --- | --- | --- | --- | --- |
| $\boldsymbol{A}_{\boldsymbol{e}}$ | Electrode area | $\boldsymbol{H}$ | Magnetic field intensity | $H$ | Pipe height |
| $\boldsymbol{D}$ | Electric displacement | $D_{e}$ | Distance between the electrode centers | $D$ | Pipe diameter |
| $\boldsymbol{E}$ | Electric field intensity | $I$ | Electric Intensity | $G_{max}$ | Maximum  conductance |
| $G$ | Conductance | $G^{*}$ | Dimensionless conductance | $G_{rel}$ | Relative conductance |
| $\boldsymbol{j}$ | Electric current density | $l$ | Characteristic length of the electrode | r | Radial coordinate |
| $\boldsymbol{j}_{\boldsymbol{D}}$ | Displacement current density | R | Pipe radius | $R_{in}$ | Inner tube dielectric radius |
| $s_{z}$ | Sensor height | $s_{w}$ | Sensor width | z | Axial coordinate |
| $\boldsymbol{r}$ | Position vector | $I_{m}(x)$ | First class modified Bessel function of m order | $K_{m}(x)$ | Second-class modified Bessel function of m order |

**Nomenclature:** **Greek symbols**

| **Symbol** | **-Definition-** | **Symbol** | **-Definition-** | **Symbol** | **-Definition-** |
| --- | --- | --- | --- | --- | --- |
| $\alpha$ | Void fraction | $\boldsymbol{\alpha}_{\boldsymbol{l}}$ | Liquid fraction | $\phi$ | Electric potential |
| $\phi_{E}$ | Electric potential at the Emitter | $\phi_{R}$ | Electric potential at the Receiver | $\sigma_{g}$ | Gas conductivity |
| $\sigma$ | Electric conductivity | $\sigma_{eff}$ | Effective conductivity | $\sigma_{w}$ | Water conductivity |
|  |  |  |  |  |  |
| $\omega$ | Angular frequency | $\delta$ | Thickness of the water film | $\theta$ | Azimuthal angle |
|  |  |  |  |  |  |
|  |  |  |  |  |  |
|  |  |  |  |  |  |

| 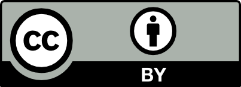 | © 2020 by the authors. Submitted for possible open access publication under the terms and conditions of the Creative Commons Attribution (CC BY) license (http://creativecommons.org/licenses/by/4.0/). |
| --- | --- |
